# Identification of LncPVT1 and CircPVT1 as prognostic biomarkers in human colorectal polyps

**DOI:** 10.1038/s41598-023-40288-1

**Published:** 2023-08-12

**Authors:** Mahsa RezaSoltani, Flora Forouzesh, Zahra Salehi, Mohammad-Reza Zabihi, Leili Rejali, Ehsan Nazemalhosseini-Mojarad

**Affiliations:** 1 Medical Genomics Research Center, Tehran Medical Sciences, Islamic Azad University, Tehran, Iran; 2 Department of Genetics, Faculty of Advanced Science and Technology, Tehran Medical Sciences, Islamic Azad University, P.O. Box: 193951495, Tehran, Iran; 3 Hematology-Oncology and Stem Cell Transplantation Research Center, Tehran University of Medical Sciences, Tehran, Iran; 4 Basic and Molecular Epidemiology of Gastrointestinal Disorders Research Center, Research Institute for Gastroenterology and Liver Diseases, Shahid Beheshti University of Medical Sciences, Tehran, Iran; 5 Department of Immunology, School of Medicine, Tehran University of Medical Sciences, Tehran, Iran; 6 Department of Cancer, Gastroenterology and Liver Disease Research center, Research Institute for Gastroenterology and Liver Diseases, Shahid Beheshti University of Medical Sciences, Tehran, Iran

**Keywords:** Cancer, Computational biology and bioinformatics, Genetics, Biomarkers

## Abstract

LncPVT1 and CircPVT1 are isoforms for the PVT1 gene and are associated with cancer progression and carcinogenesis. Our study investigated the expression of LncPVT1 and CircPVT1 in colon adenoma polyps. 40 tissues of colorectal polyps and 40 normal-adjacent tissues (NATs) were taken. The expression of LncPVT1 and CircPVT1 was evaluated through qRael-Time PCR. The relation between expression and features of clinicopathological was explored. The ceRNA network was constructed by LncPVT1 and CircPVT1 and predicted miRNAs and miRNAs targets. Further, hub nodes in this network were determined using the cytoHubba package. Over-expressed LncPVT1 and CircPVT1 were differentiated in polyp and NATs. The expression level of LncPVT1 and CircPVT1 were significantly higher in adenoma polyps than in hyperplastic polyps. The area under the curve of the ROC estimate for the LncPVT1 and CircPVT1 was 0.74 and 0.77, respectively. A positive correlation was observed between the LncPVT1 expression and CircPVT1. Three miRNAs, including hsa-miR-484, hsa-miR-24-3p, hsa-miR-423-5p, and CircPVT1, were detected as ceRNA hub nodes. In this study, expression profiles of LncPVT1 and CircPVT1 were significantly higher in precancerous polyps. In addition, based on our in silico analysis, LncPVT1, CircPVT1/miR-484, miR-24-3p, miR-423-5p/PLAGL2 axis might be involved in colon cancer development. LncPVT1 and CircPVT1 can be prescribed as warning problems as potential prognostic biomarkers in patients with pre-CRC colon polyps.

## Introduction

Cancer is a global disease affecting one out of every three men and one out of every four women^1^. Colorectal cancer (CRC) is the third most common cancer worldwide, accounting for over 8% of global cancer-related deaths. Colorectal cancer (CRC) is the third most common cancer, accounting for more than 8% of all deaths worldwide^2^. CRC is also the third largest cancer in both men and women in Asia^3^. Almost all CRCs arise from colorectal polyps^4^. Hence, CRC can be considerably prevented by identifying and eliminating adenomatous polyps^5^, Colorectal polyps are usually classified into two categories: (1) Non-neoplastic polyps (hyperplastic polyps) and (2) Neoplastic polyps (adenomatous polyps) Most colon cancers are derived from adenomatous polyps (APs), while hyperplastic polyps are not usually cancerous^6^. The majority of CRC cases arise from Adenomatous polyps^4^ Adenomatous polyps consist of adenomas and sessile serrated adenomas (SSA). Adenomas, based on histology, subtypes to tubular adenomas, tubular villous adenomas, and villous adenomas^7^. In addition, the primary medical classification of polyps is based on the definition of high-risk or low-risk polyps^6^. Based on clinical parameters, tubular adenomas larger than 1 cm, 3 or more adenomas, adenomas with villous histology or high degree of dysplasia are known as high-risk adenomas (HRA), and on the other hand, low-risk adenoma (LRA) are From 1 to 2 tubular adenomas less than 1 cm, without villous or high dysplasia Reverse splicing is a single pre-mRNA and an emerging group of cellular lncRNAs^8^.

In recent years, the role of non-coding RNAs (ncRNA) in several biological processes has been highlighted. Circular RNAs (circ RNA) and Long non-coding RNAs, types of ncRNA, have been recently discovered to be biologically involved in many cancers’ tumorigenesis, progression, and metastasis, including osteosarcoma, head and neck squamous cell carcinoma, non-small cell lung carcinoma, acute lymphoblastic leukemia, esophageal carcinoma, colorectal carcinoma, and hepatocellular carcinoma. They might be utilized as a cancer biomarker in the future^9–12^. PVT1 locus, located on chromosome 8q24.21, encodes circular PVT1 (CircPVT1) and long non-coding PVT1 (lncPVT1)^13^. They both are characterized in numerous cancers by oncogenic properties. The reason might underlie the same location of their transcription site and the cancer susceptibility locus, resulting in genome instability. Surprisingly, they are located only 53 kb downstream of the c-MYC oncogene^14^. Previous findings revealed the competitive nature of enhancers in binding to PVT1 or c-MYC promoter, thus suggesting a tumor suppressor role for lncPVT1promoter. Notably, evidence supports two separate promoters for lncPVT1 and CircPVT1 independently^15,16^ circRNAs act as cytoplasmic MicroRNA sponges (negative regulators) and participants in regulatory networks governing gene expression. A study by Wang et al. showed a significant upregulation of CircPVT1 in 92.19% of the studied CRC samples. Moreover, CircPVT1 caused the migration and invasion of CRC cells through the miR-145 sponge^17^.

LncRNAs are involved in several cellular processes, including transcriptional regulation, post-transcriptional control of mRNA, protein stability, organization of intracellular structure, and epigenetic regulation. LncPVT1 upregulation was reported previously in the study of Liu F. et al. It might inhibit apoptosis via the miR-106b-5p sponge and induce CRC progression^18^.

The present study is designed to define the close relationship between LncPVT1 and CircPVT1 in different types of polyps that can essentially initiate colon cancer. To this end, we investigate the expression of LncPVT1 and CircPVT1 genes in colon adenoma polyps using bioinformatics and experimental assays.

## Results

The clinicopathological characteristics of patients with colorectal polyps showed in Table 1.

**Table 1:** Clinicopathological characteristics of 40 patients with colorectal polyps.

	LncPVT1 (%)	Mean ± SEM	*P* value	CircPVT1 (%)	Mean ± SEM	*P* value
Age, mean (SD)						
< 50	9 (22.5%)	− 2/218 ± 2/051	0.2862	9 (22.5%)	− 1/402 ± 1/681	0.4095
> 50	31 (77.5%)			31 (77.5%)		
Gender, N (%)						
Female	20 (50%)	− 2/210 ± 1/701	0.2018	20 (50%)	2/195 ± 1/371	0.1177
Male	20 (50%)			20 (50%)		
Family history						
No	29 (36.25%)	− 2/399 ± 1/908	0.2163	29 (36.25%)	− 1/333 ± 1/572	0.4018
Yes	11 (13.75%)			11 (13.75%)		
Dysplasia						
HGD	9 (11.25)		0.4983	9 (11.25)		0.3773
LGD	11 (13.75%)			11 (13.75%)		
MGD	4 (5%)			4 (5%)		
Free D	16 (20%)			16 (20%)		
Size						
< 5	17 (21.25%)	− 2/528 ± 1/710	0.1476	17 (21.25%)	− 1/663 ± 1/407	0.2446
> 5	23 (28.75%)			23 (28.75%)		
Location						
Left side	21 (26.25%)	− 2/820 ± 1/680	0.1014	21 (26.25%)	− 2/060 ± 1/379	0.1501
Right side	19 (23.75%)			19 (23.75%)		
Polyp versus paired tissue						
Polyp	40 (50%)	− 1/923 ± 0/9097	0.0378	40 (50%)	− 2/583 ± 1/7101	0.0005
Paired tissue	40 (50%)			40 (50%)		
Pathology						
Tubular	21 (26.25%)		0.7378	21 (26.25%)		0.3382
Villous	7 (7.75%)			7 (7.75%)		
Tubulovillus	4 (5%)			4 (5%)		

### LncPVT1 and CircPVT1 expression in colorectal polyps

**Figure 1:**
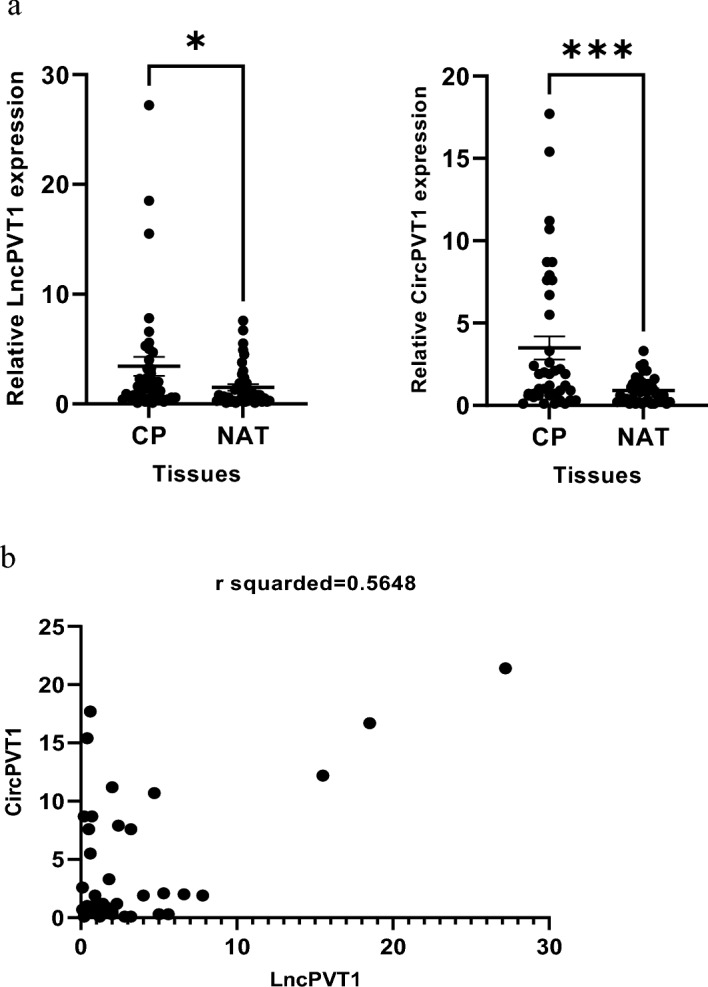
(**a**) LncPVT1 and CircPVT1 expression levels in 40 CP tissues and 40 NATs tissues were measured by qPCR. Their expression is significantly up-regulated in PC tissues compared with NATs (*P*-value: 0.0378 and *P*-value: 0.0005, respectively). GAPDH was used as an internal control. (**b**) Correlation between LncPVT1 and CircPVT1 expression levels in PC tissues had a positive relationship significantly (r = 0.5, *P*-value = 0.0001). CP: colorectal polyps, NATs: paired normal adjacent tissues. ***P < 0.001, *P < 0.05.

We investigated LncPVT1 and CircPVT1 expression in tissues of colorectal polyps and normal adjacent tissues (NATs). The results showed that LncPVT1 and CircPVT1 are significantly up-regulated in polyps tissues compared with NATs (P-value: 0.0378, 0.0005, respectively) (Fig. 1a). Furthermore, The Spearman’s correlation demonstrated that LncPVT1 expression had positive correlation with the CircPVT1 expression (r = 0.5, 95% CI 0.3074 to 0.7452, *P*-value = 0.0001) significantly (Fig. 1b). The results showed the upregulation in LncPVT1 as well as Circ PVT1 in polyps tissues.

**Figure 2:**
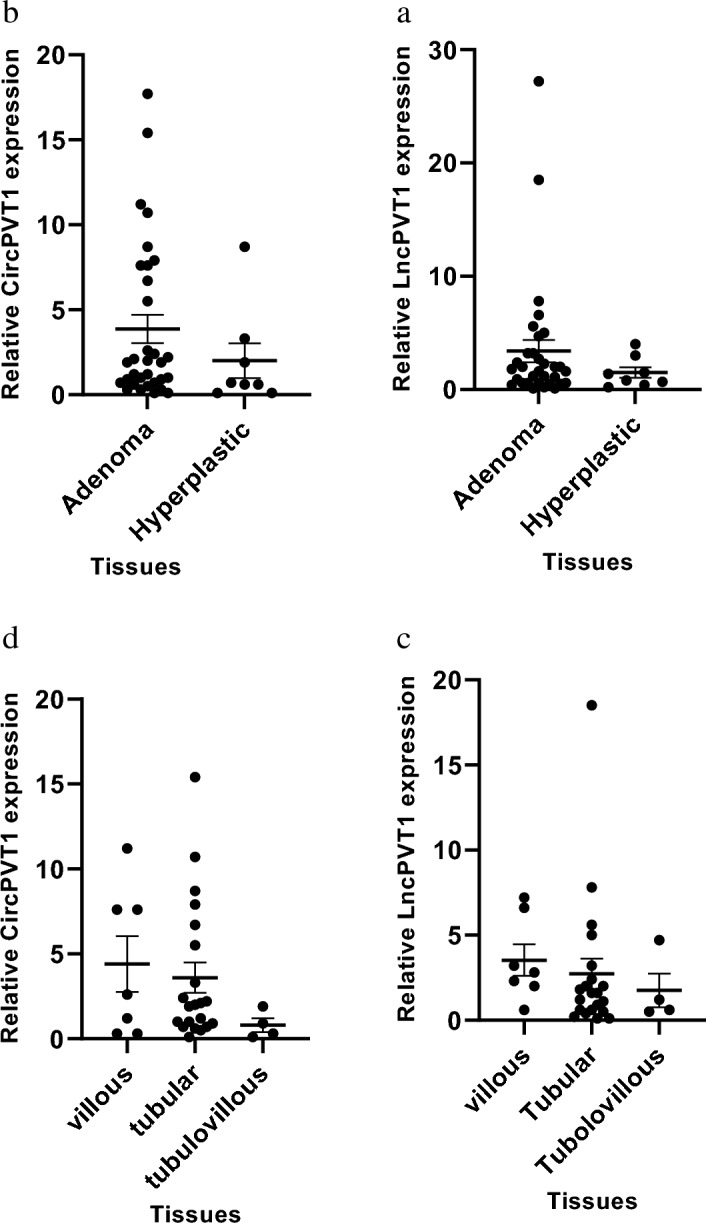
The expression levels of LncPVT1 and CircPVT1 in different types of colorectal polyps. (**a**) LncPVT1 expression levels in adenoma and hyperplastic polyps. The expression of LncPVT1 is up-regulated in adenoma polyps compared with hyperplastic, but not significantly (*P*-value 0.3489). (**b**) CircPVT1 expression levels in adenoma and hyperplastic polyp. The expression of CircPVT1 is up-regulated in adenoma polyps compared with hyperplastic, but not significantly (*P*-value 0.2901). (**c**) LncPVT1 expression levels in the villous, tubular, and tubule-villous polyp. The expression of LncPVT1 is up‐regulated in villous polyps compared with others, but not significantly (*P*-value: 0.7378). (**d**) CircPVT1 expression levels in villous, tubular, and tubule-villous polyps. The expression of CircPVT1 is up‐regulated in villous polyps compared with others, but not significantly (*P*-value = 0.3382).

Adenomatous polyps will gradually show dysplastic changes, differentiating them from hyperplastic polyps. To understand the difference in expression levels of LncPVT1 and CircPVT1 between adenomatous polyps and hyperplastic polyps, tissue samples were analyzed for the expression of these genes. However, the expression of LncPVT1 and CircPVT1 boosted in adenomatous polyps compared with hyperplastic polyps tissues; it did not demonstrate any significant growth (*P*-value 0.3489 and 0.2901 respectively (Fig. 2a,b). The results confirmed that hyperplastic polyps tend to be less malignant potential.We also investigated LncPVT1 and CircPVT1 expression in three different Adenomatous polyps, including tubular, villous, and tubulovillous, from the data in Fig. 2c,d. LncPVT1 and CircPVT1 expression are higher in samples of villous polyps than tubular and tubulovillous polyps though the increased expression was not significant. (P-value 0.7378 and 0.3382, respectively).

### Correlation of LncPVT1 and CircPVT1 expression with clinicopathological features of patients with colorectal polyps

**Figure 3:**
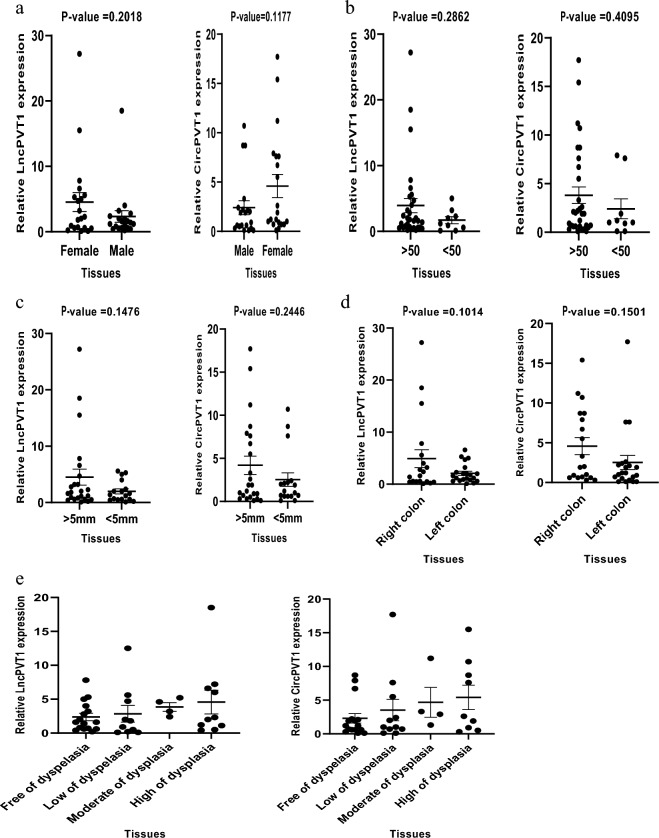
Correlation of LncPVT1 and CircPVT1 expression with clinicopathological features. The expression of LncPVT1 and CircPVT1 were compared according to different histological (**a**) gender, (**b**) age, (**c**) size, (**d**) location, and (**e**) degrees of dysplasia.

The LncPVT1 and CircPVT1 gene expression was analyzed separately in gender (male and female), age (≤ 50 and > 50), and polyp size (≤ 5 mm and > 5 mm). None of the parameters were statistically significant. The results of this investigation can be seen in Fig. 3a–c.

In this study, polyp’s locations were classified into right/proximal and left/distal-sided colon based on their extraction site. Right-sided colon (proximal) includes the cecum, ascending colon, hepatic flexure, and/or transverse colon, while the left-sided colon (distal) includes splenic flexure, descending colon, rectum colon and/or sigmoid colon. Our results showed that the expression of LncPVT increased in the right-sided colon compared to the left-sided colon, but it was not statistically significant (*P*-value = 0.1014). Similarly, the expression level of CircPVT1 increased in the right-sided colon compared to the left-sided colon, but it was not statistically significant (*P*-value = 0.1501) (Fig. 3d).

We additionally investigated the LncPVT and CircPVT1 expression in various degrees of dysplasia (high-grade, moderate-grade, low-grade) and compared them with no dysplasia samples. The results showed that LncPVT1 and CircPVT1 expression are higher in high-grade dysplasia compared to moderate and low-grade dysplasia, but no significant (*P*-value = 0.4983 and *P*-value = 0.3773 respectively). We found that the expression of LncPVT1 in high-grade dysplasia was significantly higher than in no dysplasia tissues (*P*-value = 0.4983). Also, the expression of CircPVT1 in high-grade dysplasia was significantly higher than in no dysplasia tissues (*P*-value = 0.3773) (Fig. 3e).

### Diagnostic value of LncPVT1 and CircPVT1 in colorectal polyps

**Figure 4:**
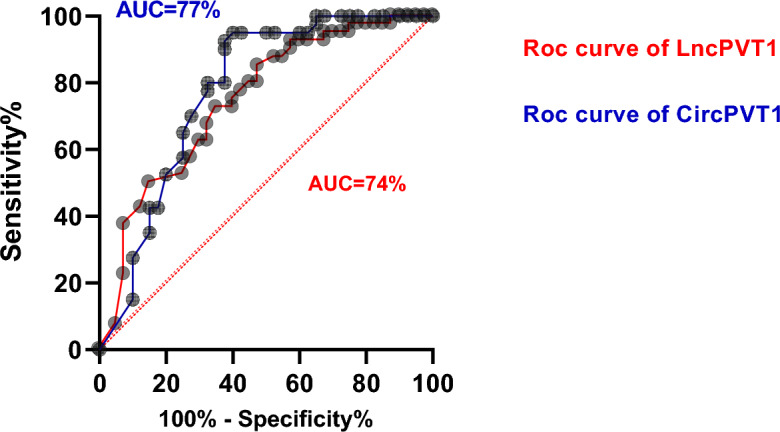
ROC curve analysis of the LncPVT1 and CircPVT1 expression, based on the area under the curve (AUC) in polyps to examine the validity of LncPVT1 and CircPVT1 genes in discriminating polyps tissues and NATs. ROC curve analysis of LncPVT1 expression showed AUC: 74% and p-value: 0.0002. ROC curve analysis of CircPVT1 expression showed AUC: 77% and *P*-value: < 0.0001. NATs: paired normal adjacent tissues.

A Receiver Operating Characteristic (ROC) curve was conducted to estimate the diagnostic value of LncPVT1 and CircPVT1 expression in colorectal polyps (Fig. 4). The results indicated that the area below the LncPVT1 ROC (AUC) curve was 0.74, with a sensitivity of 72.5% and specificity of 60%. Analysis of the CircPVT1 ROC curve revealed an AUC of 0.77 with a sensitivity of 95% and specificity of 65%.

### Interaction analysis of the CircPVT1 and LncPVT1

**Figure 5:**
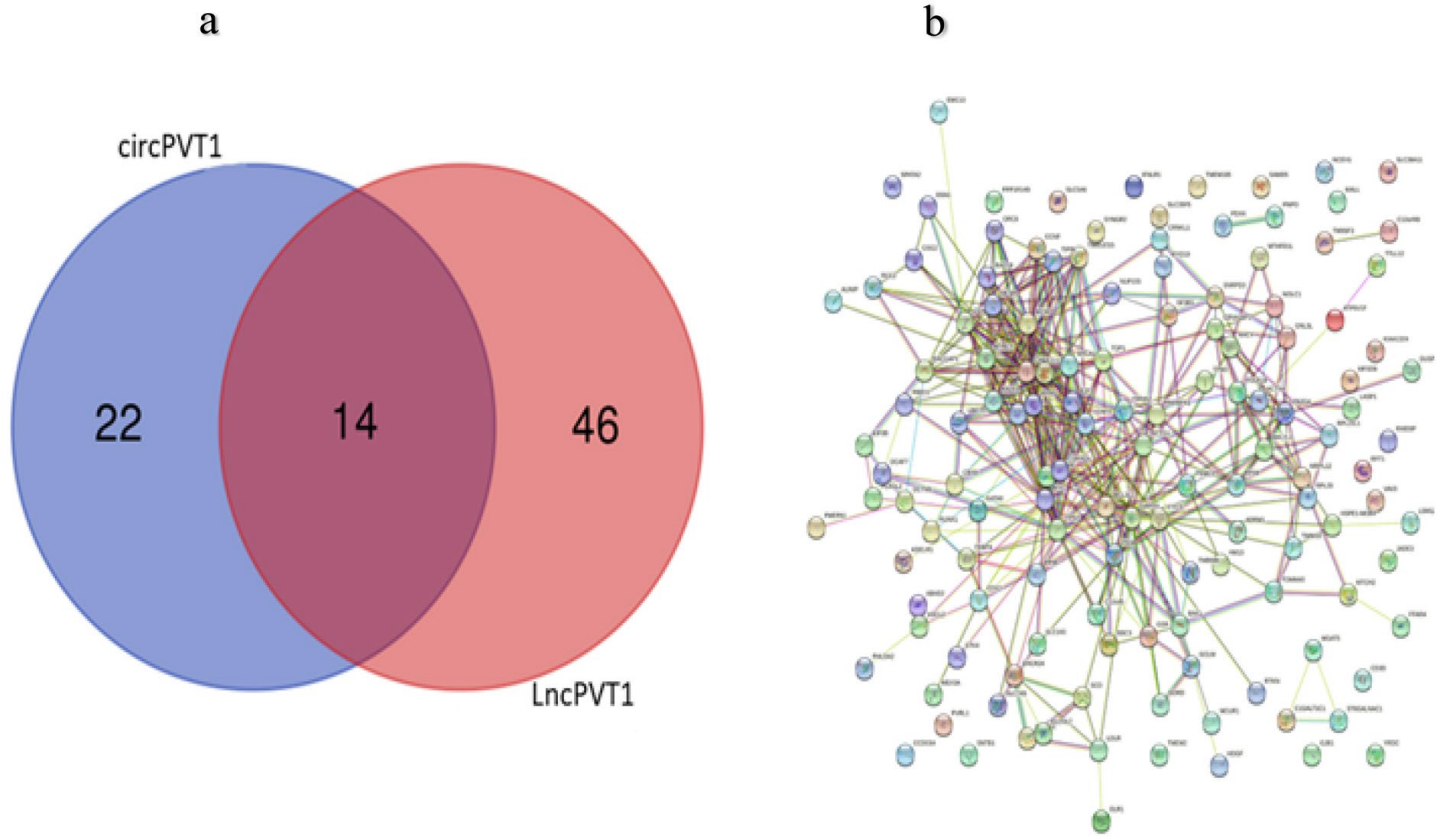
Shared miRNAs goals and related PPI network between CircPVT1 and LncPVT1. (**a**) Shared miRNAs between CircPVT1 and LncPVT1 (**b**) The PPI network is built based on the shared miRNAs targets. PPI: protein–protein interactions.

To determine the CircPVT1 and LncPVT1 miRNAs sponging function, we constructed theProtein–protein interaction (PPI) network targeted shared miRNAs between CircPVT1 and LncPVT1. 36 miRNAs sponged by CircPVT1 and 60 miRNAs sponged by LncPVT1 were detected. Both CircPVT1 and LncPVT1 were targeted by 14 shared miRNAs (*hsa-miR-4733-3p*, *hsa-miR-423-5p*, *hsa-miR-4711-5p*, *hsa-miR-4650-5p*, *hsa-miR-484*, *hsa-miR-3155a*, *hsa-miR-24-3p*, *hsa-miR-3190-5p*, *hsa-miR-3184-5p*, *hsa-miR-605-5p*, *hsa-miR-3926*, *hsa-miR-3195*, *hsa-miR-3155b*, *hsa-miR-1825*) (Fig. 5a). According to experimental data from mirTarbase, these 14 miRNAs interact with 2671 targets, 718 of which interact with over two miRNAs. Of those, 139 targets are overexpressed in COAD and READ (Supplementary file). To investigate the potential mechanisms underlying the progression of colorectal polyps to COAD and READ, we selected 139 miRNA targets for constructing the PPI network (Fig. 5b).

### Functional enrichment analysis

**Figure 6:**
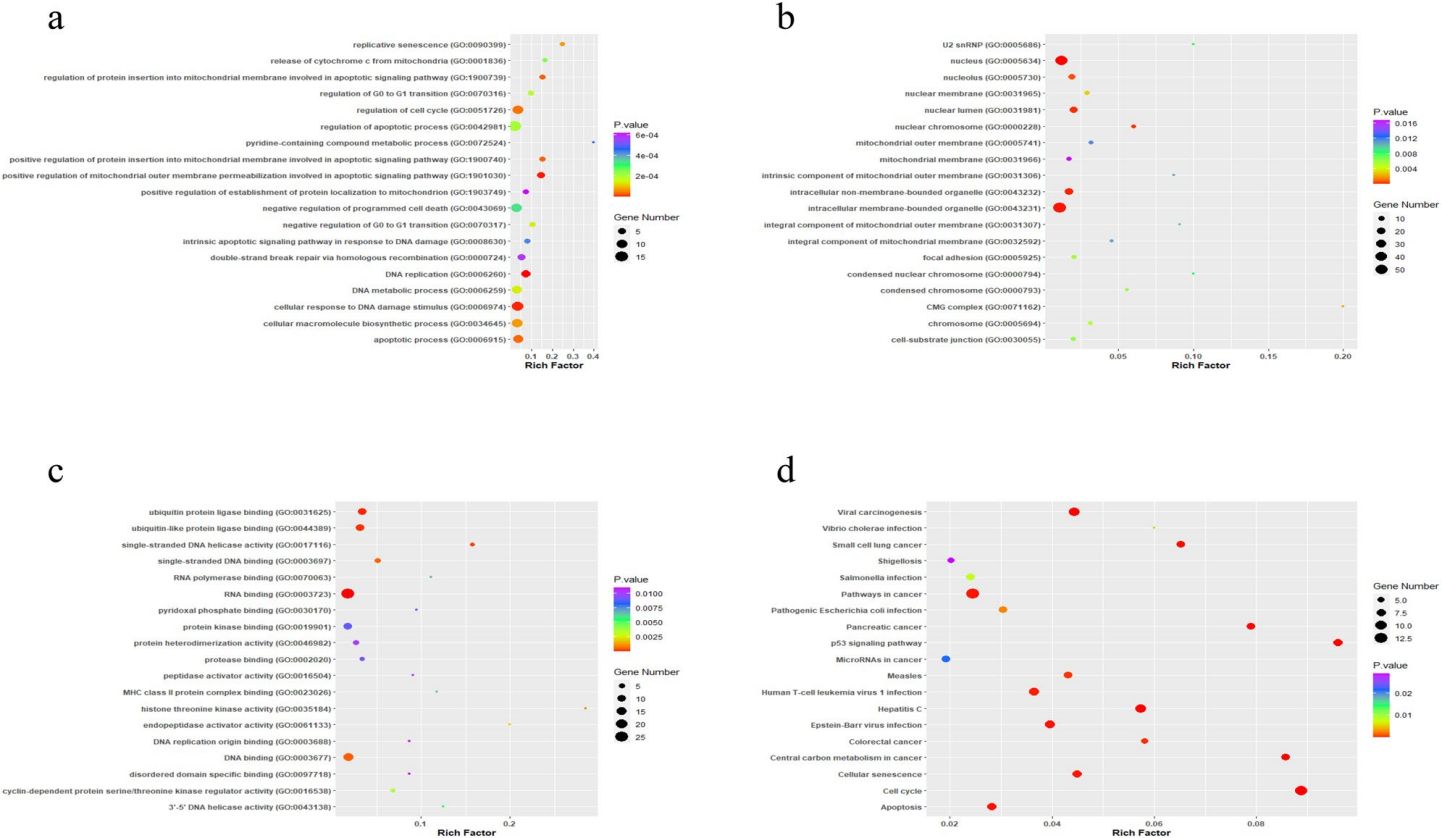
Enrichment analysis of proteins involved in the protein–protein interactions. (**a**) BP of GO terms. (**b**) CC of GO terms. (**c**) MF of GO terms. (**d**) KEGG pathways. PPI: protein–protein interactions, BP: Biological processes, CC: Cellular components, MF: Molecular functions.

**Figure 7:**
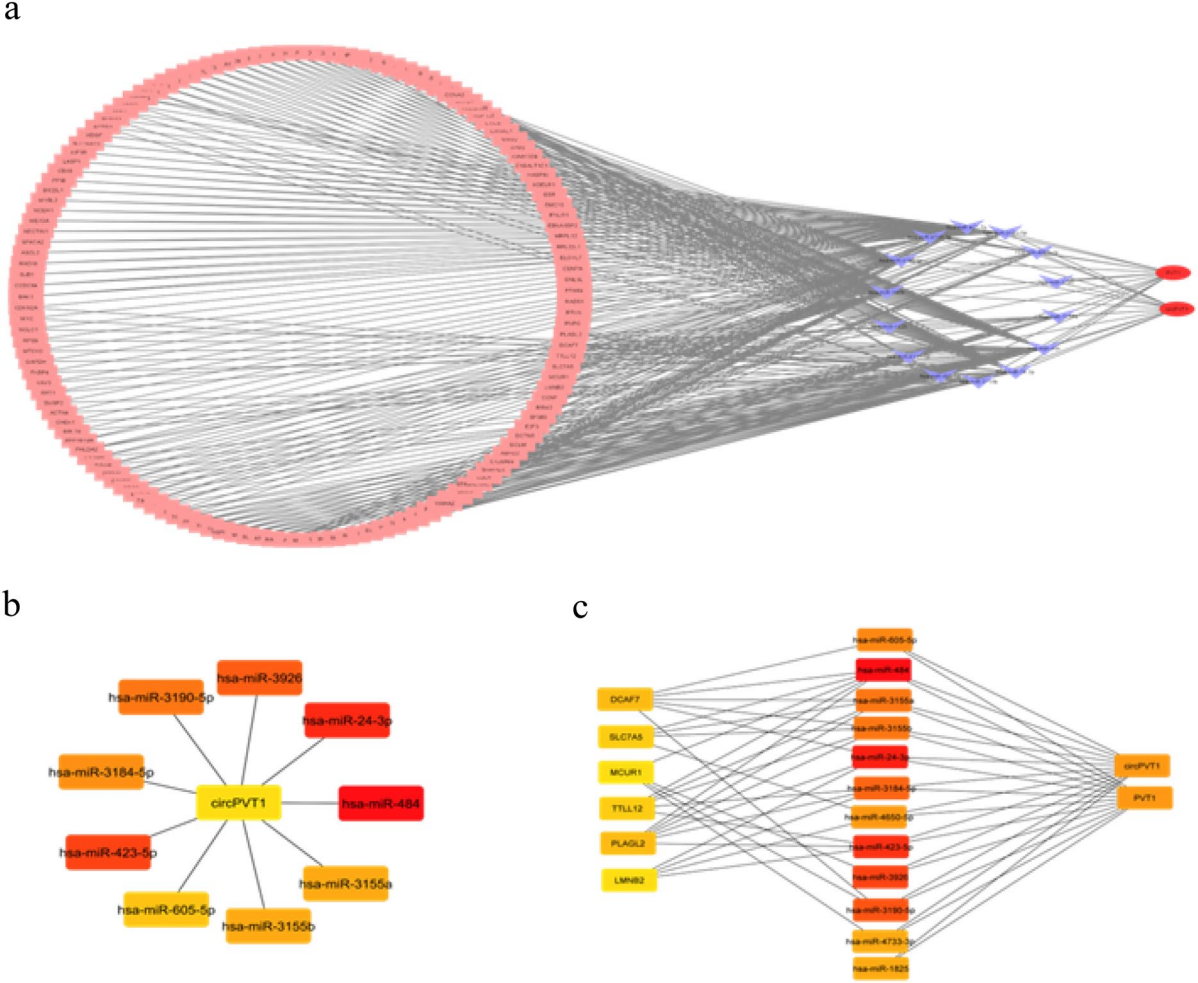
The ceRNA network and hub nodes. (**a**) The ceRNA network was constructed by CircPVT1, LncPVT1, 14 shared miRNAs, and 139 selected miRNA targets. (**b**) The 10 hub nodes identified by cytoHubba based on the degree method compromising CircPVT1 and miRNAs. (**c**) Top 20 hub nodes identified by cytoHubba based on degree method, including CircPVT1 and miRNAs, and mRNAs.

To further decipher the potential role of PPI created by miRNA targets, we performed GO and KEGG functional enrichment analyses of all these proteins. The 20 most abundant GO terms and KEGG pathways are shown in Figure 7. DNA replication was The most enriched function in terms of rich factor score and P-value in the BP category. (GO: 0006260, RF = 0.07), positive regulation of mitochondrial outer membrane permeabilization involved in apoptotic signaling pathway (GO: 1901030, RF = 0.14), cellular response to DNA damage stimulus (GO: 0006974, RF = 0.03), regulation of protein insertion into mitochondrial membrane involved in apoptotic signaling pathway (GO: 1900739, RF = 0.15). The nucleus (GO: 0005634, RF = 0.01), intracellular membrane-bounded organelle (GO: 0043231, RF = 0.01), intracellular non-membrane-bounded organelle (GO: 0043232, RF = 0.01), nuclear lumen (GO: 0031981, RF = 0.02) were the most highly enriched items in the CC category. Molecular function categories were mainly enriched in RNA binding (GO: 0003723, RF = 0.01), ubiquitin protein ligase binding (GO: 0031625, RF = 0.03), ubiquitin-like protein ligase binding (GO: 0044389, RF = 0.03), single-stranded DNA helicase activity (GO: 0017116, RF = 0.15), DNA binding (GO: 0003677, RF = 0.01), single-stranded DNA binding (GO: 0003697, RF = 0.05). Pathways in cancer (RF = 0.02), Colorectal cancer (RF = 0.05), MicroRNAs in cancer (RF = 0.01), and p53 signaling pathway (RF = 0.09) were enriched among the top 20 KEGG pathways Fig. 6.

### CeRNA network analysis

Additionally, CircPVT1 and LncPVT1, 14 shared miRNAs, and 139 miRNAs targets were utilized to construct the ceRNA network to identify hub genes associated with the disease progression (Fig. 7a). The 10 hub nodes identified by cytoHubba based on degree method, including *hsa-miR-484* (score: 67), *hsa-miR-24-3p* (score: 52), *hsa-miR-423-5p* (score: 46), *hsa-miR-3926* (score: 42), hsa-miR-3190-5p (score: 41), hsa-miR-3184-5p (score: 33), hsa-miR-3155a (score: 21), *hsa-miR-3155b* (score: 21), *hsa-miR-605-5p* (score: 15), *CircPVT1* (score: 14) (Fig. 7b). Moreover, in 20 hub nodes, some miRNAs targets enriched, including *PLAGL2* (score: 6), *DCAF7* (score: 6), *TTLL12* (score: 5), *SLC7A5* (score: 5), *MCUR1* (score: 4), *LMNB2* (score: 4) (Fig. 7c). Based on UALCAN online database, miR-484 downregulated in COAD tissue (*P*-value = 4.80E−04) and READ tissue (*P*-value = 2.41E−12), miR-24-3p downregulated in COAD and READ tissue but not significant, miR-423-5p downregulated in COAD tissue (*P*-value = 3.94E−06) and READ tissue (*P*-value = 1.63E−12), and PLAGL2 up-regulated in COAD tissue (*P*-value < 1E−12) and RAED tissue (*P*-value < 1E−12).

## Discussion

Colorectal cancer is a major cause of global mortality. Early detection is essential to enhance overall survival, reduce progression without disease, and reduce the risk of recurrence. Biomarkers are key in early disease identification and can help predict disease progression and treatment response^19^. Several studies conducted by different researchers have established the crucial role of LncRNAs in physiological and pathological processes through various mechanisms^20,21^. It has been widely reported that PVT1 promotes tumor growth in different types of cancer. Ovarian cancer cells are affected by PVT1 through miR-370 sponges. As reported by Angel and colleagues, PVT1 is a novel long non-coding RNA highly expressed in gastric cancer tissue^22^. PVT1 is also highly expressed in colorectal cancer tissue, and downregulating PVT1 reduces malignant behaviors, including cell proliferation, migration, and invasion^23^. Overexpression of PVT1 by suppression of MYC protein levels leads to the proliferation of leukemic cells in acute promyelocytic leukemia^24^. Furthermore, cancers with high PVT1 expression have a poor prognosis and mortality rate^25^. According to research, overexpression of PVT1 promotes malignant behavior in ovarian cancer cells, while downregulation of PVT1 inhibits it, suggesting that PVT1 may play an important role in cancer progression. It appears that the treatment is working. Breast cancer's malignant behavior may also be regulated by LncRNA epigenetically regulating their downstream genes^26^. Based on the mentioned reports, malignant cancer cells are regulated by Lnc RNAs through different mechanisms. Recently, LncRNAs function as competitive endogenous RNAs (ceRNA) to inhibit miRNAs and protect target mRNAs from degradation^27^. miR-370 binds specifically to PVT1, and FOXM1 protein regulation is maintained by PVT1 overexpression. PVT1 potentially regulates FOXM1 protein levels through binding epigenetically to FOXM1 protein, and promotes FOXM1 mRNA translation by using miR-370. So, the depletion of both PVT1 and miR-370 decreases FOXM1 protein levels. Accordingly, PVT1 can function as an oncogenic factor in ovarian cancer cells by regulating the FOXM1 protein level. It has also been shown that PVT1 epigenetically and post-transcriptionally regulates FOXM1, which can provide new insights into the complex regulation of lncRNA in cancers^28^. Zhao et al. indicated that lncRNA PVT1 acts as a sponge for miR-448 in order to promote the pancreatic ductal adenocarcinoma development. Similarly, Shen et al.^29^ recently showed that lncRNA PVT1 could increase FSCN1 gene expression in order to enhance esophageal cancer (EC) cell migration and invasion, and also to induce apoptosis by binding to miR-145. Research has shown that LncRNAs can also play an important role in tumorigenesis and development. Furthermore, He et al.^30^ found that PVT1 was overexpressed in human CRC tissues. Recently, the accumulation of studies has shown that PVT1 plays a major role in CRC, but the exact mechanism is not clear^30,31^. In recent studies, PVT1 was strongly related to poor prognosis and poor clinical-pathological characteristics. In addition, cell growth and invasion, as well as the ability to migrate, were inhibited in CRC cells following the reversal of PVT1. This data indicate that PVT1 is an oncogene lncRNA and is associated with increased risk of late-stage tumor invasion and poor overall survival in various cancers, including CRC. It may also be associated with chemoresistance to drugs commonly used to treat CRC. The relative stability of PVT1 compared to endogenous RNases may make it easier to use as a biomarker.

Nevertheless, developing PVT1 as a diagnostic, prognostic or therapeutic biomarker is difficult^32^. In short, PVT1 cRNA expression is enhanced in CRC cells and tissues. In patients with CRC, increased expression of PVT1 is associated with poor prognosis and more severe clinical and pathological features. They offer new insights into the underlying mechanism of disease progression and suggest that PVT1 could be used as a potential prognostic biomarker and promising therapeutic target for the disease.

Chai et al. studied patients with colorectal cancer in 2018. They reported that LncPVT1 increases the expression of RUNX2 (the most specific transcription factor in the mesenchymal stem cells differentiation to osteoblasts) by using sponges and inhibiting miR-455, which promotes the development of this cancer^33^. Lee and colleagues also reported that CircPVT1, compared to the adjacent normal mucosal tissues, has an increased expression in colorectal cancer^34^. The present study showed that the expression of two Lnc genes, PVT1 and CircPVT1, compared to peripheral tissues of polyps, has increased significantly in the tissues with adenomatous polyps. Increasing expression of Lnc genes can not only cause the progression from adenomatous polyp to malignancy and colorectal cancer but also can be useful in diagnosis, prognosis, and treatment. According to the present study, the expression of LncPVT1 and CircPVT1 genes increased, not statistically significant, in samples with severe dysplasia compared to samples without dysplasia. Also, the present study investigated the LncPVT1 and CircPVT1 genes' expression levels in three adenoma polyp groups, including villous, tubular, and tubulovillous. The results showed that the expression levels of both genes increased in the villous group, compared to the tubular and tubulovillous groups, and this indicates the villous group's potential to cause malignancy. This increased expression in the villus group can indicate that they have a greater potential to cause malignancy. In 2018, Chai et al. also reported a significant relationship between polyp size, disease severity, and the location where the intestine is involved, which is associated with invasive cancer-increasing risk^33^. Researchers in 2020 reported that patients with RSCC were more likely than patients with LSCC to exhibit severe tumor stage, increased tumor size, often benign tumors, and increased lymphovascular invasion at the physiological level^35^. In the present study, LncPVT1 and CircPVT1 genes were expressed further in the right region of the colon than in the left. However, there was no statistically significant relationship between the gene expression levels in the two regions. In people under 50 years of age, 12% in women and 24% in men have been reported, and in those over 80 years of age, it has increased by 27% for women and 4% for men, respectively^36^. Yosimek et al. showed in 2016 that adenoma is more common in men between 50 and 60 years old^37^. In 2013, based on Corelli and colleagues' research, LnvPVT1 and CircPVT1 expression in this study were investigated according to gender and age^38^. The results showed that LncPVT1 and CircPVT1 genes in women over 50 were more increased than in men under 50, but it was not statistically significant. Our results showed that the changes in these two genes' expression do not depend on gender and age. Possibly, the non-significance of the results could be in relation to the little number of samples and also the number inequality of the two studied groups. Kurkmaz, Kandir and Akaya, In 2016, reported that the greater the polyp diameter (2 cm or larger) grows, the more the risk of dysplasia malignancy increases^37^. According to the present study, polyps with a greater size of 5 mm demonstrated more changes in LncPVT1 and CircPVT1 gene expression compared to those smaller. However, it was statistically nonsignificant. In the analysis of the ROC curve for the LncPVT1 gene, it has been shown that the area under the graph (AUC) is equal to 0.74, and the sensitivity and specificity of LncPVT1 for polyp detection were 72.5 and 65%, respectively, but it is not statistically significant. *P*-value = 0.0002). In the examination of the ROC curve for CircPVT1 gene, it has been shown that the area under the graph (AUC) is 0.77. The sensitivity and specificity of CircPVT1 for polyp detection were 95% and 60%, respectively. It was statistically significant (*P*-value =  < 0.0001).

To determine important Circ and Lnc/miRNA/mRNA axis that might play a key role in the progression of adenoma polyps to colorectal carcinoma, the ceRNA network was constructed and analyzed. The top three hub nodes detected from ceRNA were hsa-miR-484, hsa-miR-24-3p, and hsa-miR-423-5p. Chai et al. demonstrated hsa-miR-484 downregulation in colorectal carcinoma tumor tissues compared with matched normal tissues^39^. Accumulating data indicated LncRNAs sponging function on hsa-miR-484 in CRC development^40^. Gao et al. represented hsa-miR-24-3p downregulation in CRC tissues and the effects of hsa-miR-24-3p inhibition on cell proliferation, migration, and tumor invasion^41^. Jia et al. indicated hsa-miR-423-5p downregulation in colon malignant tissues and cancer cell lines and suggested hsa-miR-423-5p as a tumor suppressor in colon cancer^42^. Together, these studies validate the downregulation of hsa-miR-484, hsa-miR-24-3p, and hsa-miR-423-5p enriched from our bioinformatics analysis involved in CRC development,—all three miRNAs sponged by CircPVT1 and LncPVT1 cause downregulation of the three miRNAs in CRC.

Moreover, based on our analysis, PLAGL2 was targeted by the three miRNAs. Li et al. demonstrated the upregulation of PLAGL2 in CRC tissues and suggested PLAGL2 as an oncogene involved in CRC development^43^. To these data, LncPVT1, CircPVT1/miR-484, miR-24-3p, miR-423-5p/PLAGL2 axis might be involved in CRC development.

According to KEGG data, the possible pathways involved in CRC development were enriched, including the p53 signaling pathway, cancer-related pathways, CRC, cancer-related microRNAs, central carbon metabolism in cancer, and apoptosis. Moreover, some intestine and colon-related infections, including pathogenic Escherichia coli infection, Salmonella infection, and Shigellosis were enriched. Various studies represent E. coli procarcinogenic properties in murine models^44^. Raisch et al. indicated E. coli colonization on the mucosa of colon cancer patients^45^. Mughini-Gras et al. showed that patients diagnosed with severe salmonellosis have an increased risk of developing cancer in the ascending/transverse parts of the colon^46^. Shigella occurs in the tumor microenvironment during the tumorigenesis process. Shigella is a potentially pro-oncogenic pathogen^47^. This study includes limitations such as the limited number of samples and lack of investigation of other CircRNAs related to this gene.

## Conclusions

In many types of cancer, the expression of CircPVT1 and LncPVT1 increases and is related to various clinical features, including survival and lymph node metastases, and CircPVT1 enhances cancer cell growth, proliferation, cell migration, invasion, and drug resistance. LncPVT1 and CircPVT1 act as sponges for tumor suppressor miRNAs with oncogenic properties, including hsa-miR-484, hsa-miR-24-3p, and hsa-miR-423-5p (and participate in immune cell differentiation and function). Regulation of PVT1/CircPVT1 in cells through genomic amplification, rearrangement, or increased transcription provides an advantage for cancer cell proliferation. Since intestinal polyps can lead to life-threatening adenocarcinoma, the quick and accurate diagnosis and predictor for precancerous polyps might save lives. Our findings support biomarker and drug-gable target potential for PVT1 and CircPVT1, although more research is still needed into this area.

## Methods

### Ethics statement

This study was approved by the ethics committee of the Azad Islamic University of Medical Sciences (ethics review report number: IR.IAU.PS.REC.1400.423), and written informed consent was obtained from all participants. All experiments were performed by relevant guidelines and regulations. The information regarding the number, size, location, and histological characteristics of the reference polyps was extracted from the endoscopic and pathology reports. All participants were provided thorough study information and signed an informed contest.

### Samples selection and patient’s criteria

This study obtained polyp samples from patients referred to Talegani Hospital. Based on inclusion and exclusion criteria, 40 colonic polyp biopsies and 40 adjacent normal specimens were obtained. Patients receiving chemotherapy and taking certain drugs (anti-inflammatory) were excluded from this study.

### RNA extraction, cDNA syntheses, and qRT-PCR

**Figure 8:**
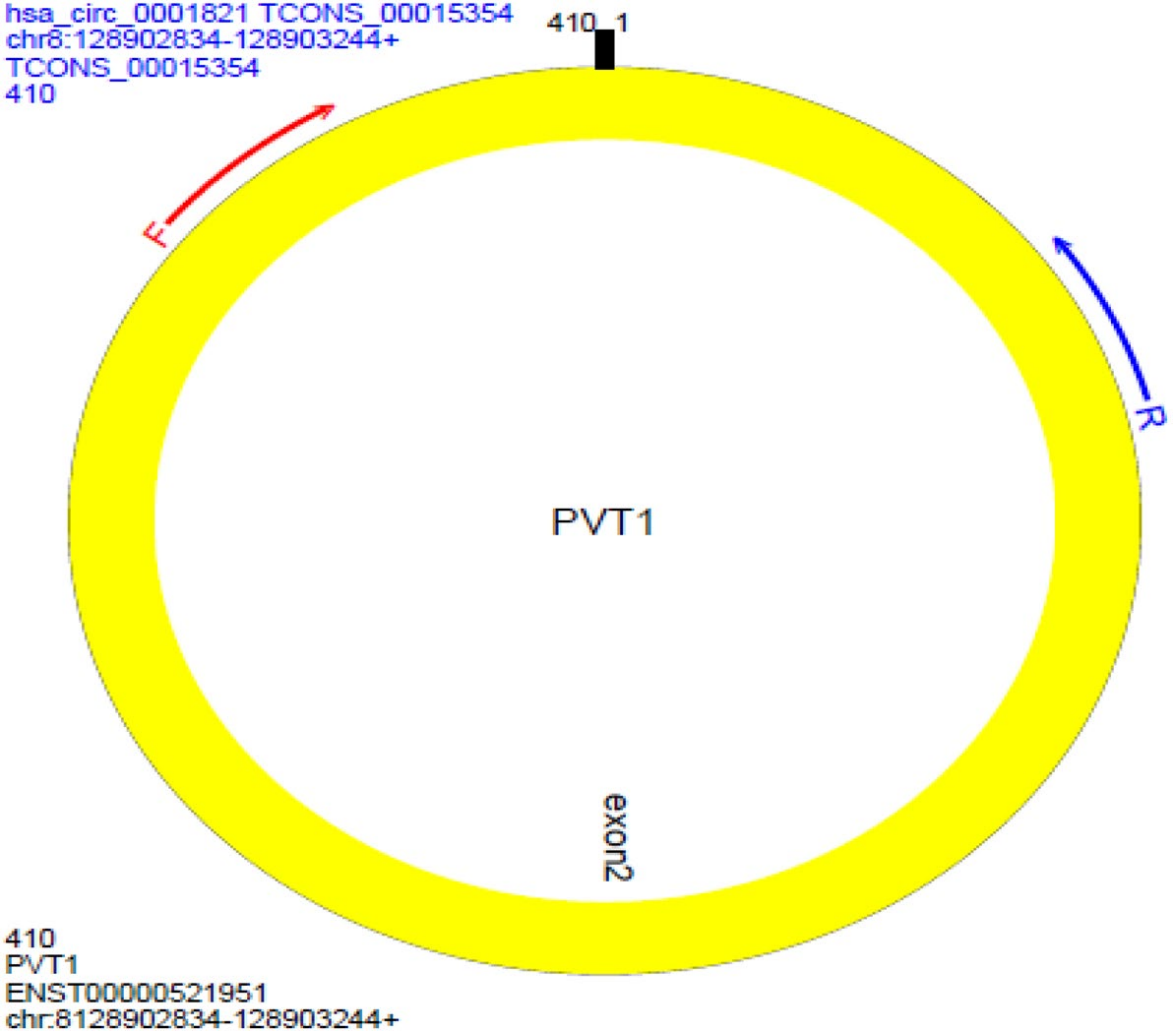
Divergent primers and the black vertical line represents the CircPVT1 back-spliced junction Primer pattern of CircPVT1. The blocks represent exon 2. Red and blue arrows represent.

The prepared tissue was stored at − 80 °C. Total RNA extraction was then performed using the TRIzol reagent kit (YeKta Tajhiz Azma kit, Cat. No. YT9065, Tehran, Iran) according to the manufacturer's instructions. Both the quality and quantity of the extracted RNA were then assessed using a NanoDrop 1000 spectrophotometer (NanoDrop Technologies, Wilmington, DE, USA). cDNA was then synthesized in a final volume of 13.4 μl using a kit (TaKaRa Kit, Cat. No. RR037A, Otsu, Shiga, Japan). Primers for LncPVT1, CircPVT1 (Fig. 8), and Glyceraldehyde-3-phosphate dehydrogenase (GAPDH) as a housekeeping gene, were presented the following: Forward LncPVT1: 5′-CTTCCAGTGGATTTCCTTGC-3′, Reverse LncPVT1: 5′-CATCTTGAGGGGCATCTTTT-3′, Forward CircPVT1: 5′-GACTCTTCCTGGTGAAGCATCTGAT-3′, Reverse CircPVT1: 5′-TACTTGAACGAAGCTCCATGCAGC-3′, Forward GAPDH: 5′-CCTGCACCACCAACTGCTTA-3′, Reverse GAPDH: 5′-GGCCATCCACAGTCTTCTGG-3′.

Quantitative Reverse Transcription PCR (qRT-PCR) was performed using Applied Biosystems 7500 version 1 (ABI, Foster City, CA, USA) using SYBER Premix Ex TaqII (TaKaRa kit, catalog number RR820A, Otsu, Shiga, Japan). The real-time PCR conditions were: 95 °C for 15 min, followed by 40 cycles of 95 °C for 10 s and 60 °C for 30 s, and 72 °C for 35 s.

### Protein–protein interaction network

To construct the protein–protein interaction network (PPI) network, the microRNAs sponged by both CircPVT1 and LncPVT1 were identified using circinteractome^48^, and miRDB databases^49^. Then, Bioinformatics and Evolutionary Genomics web tool (https://bioinformatics.psb.ugent.be/webtools/Venn/) was utilized to find shared miRNAs sponged by CircPVT1 and lncPVT1. Subsequently, the mirTarbase database^50^ was employed to detect the targets of shared miRNAs. The PPI network of shared miRNAs targets was determined using the STRING database^51^.

### Enrichment analysis

Over-expressed miRNA targets in COAD and READ have been selected as a query for the Enrichr database^52^. Data on biological processes (BP), molecular functions (MF), cellular components (CC), and KEGG pathways were extracted. Next, records with a *P*-value < 0.05 were selected, and the higher features based on the *P*-value were candidates for display by the packet “ggplot” in the R programming language^53^.

### Competitive endogenous RNA (CeRNA) network

Since polyp adenocarcinoma predisposes patients to colorectal cancer and rectum adenocarcinoma, targets that interact with more than two shared miRNAs selected to evaluate their significant overexpression in colon adenocarcinoma (COAD) and rectum adenocarcinoma (READ) by GEPIA2 (http://gepia2.cancer-pku.cn)^54^. Then, the CeRNA network was constructed by CircPVT1, LncPVT1, and the overexpressed mRNA targets in COAD and READ. The minimum requirement for interaction was 0.4. Competitive endogenous RNA (ceRNA) network constructed by Cytoscape^55^ and hub nodes identified by cytoHubba package^56^ based on degree method. Further, the UALCAN database (https://ualcan.path.uab.edu/)^57^ was used to provide further validation regarding the detected core genes.

### Fold change calculations and statistical analysis

LncPVT1 expression and CircPVT1expression assessments between tumoural, adjacent normal and normal tissue groups were performed, and the fold change or RQ validation was determined by using the (2 − ΔΔCT) method and by use of nonparametric Kruskal–Wallis test and Mann–Whitney where ever needed. All samples were double-checked. The log-rank test was performed to determine statistical significance. RQ values observed a significant relationship between the target and control groups. RQ < 0.5 was interpreted as a decrease in the expression level, and RQ value > 2 assign as an increase in gene expression. Receiver Operating Characteristic (ROC) curves for early and late stages were drawn, and mean RQs were calculated.

### Supplementary Information

Supplementary Information.

## Data Availability

The data presented in this study are available on request from the corresponding author.
